# Virtual Summer Undergraduate Mentorship Program for Students Underrepresented in Medicine Yields Significant Increases in Self-Efficacy Measurements During COVID-19 Pandemic: A Mixed Methods Evaluation

**DOI:** 10.1089/heq.2021.0060

**Published:** 2021-09-30

**Authors:** Cara Stephenson-Hunter, Stacey Franco, Alicia Martinez, A. Hal Strelnick

**Affiliations:** ^1^ Department of Family and Social Medicine, Albert Einstein College of Medicine, Bronx, New York, USA.; ^2^ Office of Diversity Enhancement, Albert Einstein College of Medicine, Bronx, New York, USA.

**Keywords:** underrepresented in medicine, premedical education, COVID-19, Social Cognitive Career Theory, diversity in medicine, virtual mentorship

## Abstract

**Purpose:** The barriers that Black and Hispanic/Latinx students underrepresented in medicine (URiM) face while seeking biomedical careers have been identified, including lack of career preparation and social support. Yet it is unclear how the COVID-19 pandemic has impacted their decisions and progress toward their occupational goals.

**Methods:** Adapting to the precautions necessitated by the COVID-19 pandemic, the authors conducted a mixed-methods evaluation of our 2020 virtual summer URiM biomedical mentoring program, using both quantitative and qualitative pre- and post-program questionnaires to measure the students' perceptions of their preparation and self-efficacy for applying to health professional schools.

**Results:** Themes were extracted from qualitative data through thematic analysis. When students were asked how the COVID-19 pandemic affected them, two themes and subthemes emerged: (1) loss of supportive environment, (1.1) challenging learning environment, (2) derailed or uncertain future, and (2.2) COVID-19 hitting home. When students were surveyed about their online experience at the end of the program, the themes (3) supportive community and (4) inspired and reinforced goals appeared. In addition, quantitative data examined through statistical analysis revealed that the students' career self-efficacy improved significantly after program participation.

**Conclusion:** Our results indicate that the COVID-19 pandemic has further challenged URiM students with pre-existing career obstacles. The outcomes also demonstrate that it is possible to virtually provide URiM students with socioemotional support and increase their career self-efficacy. Overall, frequent evaluations are encouraged to better understand the dynamic challenges of URiM students, improve the design of health career “pipeline” programs, and diversify the physician workforce to address racial health inequities.

## Introduction

Many scholars agree that health equity can be better achieved through a health workforce that reflects our racial and ethnically diverse communities in the United States.^1–3^ Although this strategy appears promising for combating health disparities, racial concordance in medicine would be difficult to achieve due to the disproportional makeup of the current physician workforce. While the U.S. population is 13% Black and 18% Hispanic/Latinx, these groups currently only constitute 2.6% and 5.8% of physicians, respectively, so they are, therefore, underrepresented in medicine (URiM).^4,5^ URiM students on the biomedical career track face a multitude of barriers, making it difficult to remain on track and matriculate into biomedical degree programs.^1,6^ Challenges with “gateway” science courses, lack of career knowledge and preparation, and less social support (including mentoring), are well-known barriers for URiM students, contributing to the lack of diversity observed in the biomedical field.^7–9^ “Pipeline” programs were created to address the disadvantages faced by URiM students and facilitate their entry into the health and biomedical professions.^10–14^ Summer pipeline programs are intended to improve diversity in the medical and biomedical fields through brief, intensive interventions focused on science enrichment, clinical exposure, and mentorship.^10,15,16^

Albert Einstein College of Medicine and its university hospital, Montefiore Medical Center, in the Bronx NY, host five biomedical pipeline programs for URiM and disadvantaged undergraduate students each summer.^17^ The 2020 pandemic resulted in the closing of college campuses and the suspension of many summer workforce and educational enrichment opportunities. To continue supporting URiM students during the global pandemic, the five pipeline programs collaborated on the development and launch of the Bronx Health Opportunities Partnership @ Einstein (Bronx HOPE) Virtual Summer Program. Although the pandemic precluded traditional face-to-face mentoring, we adapted our existing curricula for virtual learning and piloted a 6-week, virtual mentorship program for 46 URiM and disadvantaged undergraduates.

A majority of our students come from underserved, low socioeconomic communities, including the Bronx. The Bronx has the lowest high school and college graduation rates and the greatest incidence of poverty and premature deaths of all New York counties^18,19^ and the highest COVID-19 hospitalization and mortality rates of New York City's five boroughs.^20^ In addition, historically disadvantaged groups have been hit hardest by the negative educational outcomes associated with the pandemic; this includes the thousands of low-income, minority, pre-health college students already facing academic and social barriers while on the path to biomedical careers.^21,22^ During the COVID-19 pandemic, research has found that college students from low socioeconomic backgrounds are more than 50% more likely to delay graduation and non-white students are 70% more likely to change their majors compared to their more affluent peers.^23^

Building upon Bandura's Social Cognitive Theory, which posits how the intersection of intrinsic and extrinsic factors influence psychosocial functioning, Lent et al. established the Social Cognitive Career Theory (SCCT) to explain how these factors interact during an individual's career development process.^24–27^ SCCT describes how academic and career interests and decisions are formed and how occupational persistence and performance are achieved.^27–29^ With its social distancing and remote learning, how the 2020 pandemic affected URiM students' career goals and decisions is unknown as is the impact of a virtual program on these career factors. Thus, the objectives of this evaluation are to (1) describe the intermediate outcomes of an innovative virtual pipeline program for pre-health URiM undergraduates during the COVID-19 pandemic, (2) explore URiM undergraduate students' experiences and perspectives during the 2020 COVID-19 pandemic and how their academic/career goals were affected, and (3) assess their perceptions of the virtual program and influence on their career decisions and goals.

## Methods

### Participants

Eighty-four undergraduate students who applied to our five 2020 summer Einstein/Montefiore programs were invited by email to join the Bronx HOPE Virtual Summer Program, as well as to complete a self-assessment survey on the content of our 6-week program. Sixty-three students completed the survey; 55 expressed interest in joining; and 46 enrolled (i.e., completed program contract/requirements). See Table 1 for their demographic characteristics.

**Table 1.** Sex, Race/Ethnicity, Area of Residence, and Academic Status of the 46 Bronx Health Opportunities Partnership at Einstein Virtual Summer Program Students

	No.	Percentage
Sex		
Female	38	83
Male	8	17
Race and ethnicity		
Hispanic/Latinx	19	41
African American/Black	19	41
Asian	6	13
White	2	5
Area of residence		
NYC boroughs	29	63
New York-outside of NYC^a^	7	15
Out of state^b^	10	22
First-generation student^c^		
Yes	20	43
No	21	46
Unknown^d^	5	11
Level of education as of Fall 2020		
Freshman or sophomore	6	13
Junior	22	48
Senior or recent grad	18	39

^a^ Locations include Westchester, Long Island, Orange County, Rochester, and Buffalo.

^b^ Locations include New Jersey, Georgia, Louisiana, Ohio, Colorado, Maryland, California, Texas, and Pennsylvania.

^c^ Defined as a first-generation individual within a family to go to college/pursue a 4-year degree.

^d^ Student did not complete this question on the pre-survey administered before the start of the program.

NYC, New York City.

### Program description

The Bronx HOPE Virtual Summer Program was designed to meet URiM student needs, including mentorship, improvement of professional/intrapersonal skills, knowledge on applying to graduate biomedical programs, and exposure to scientific topics relevant to medical careers. The American Association of Medical Colleges' Core Competencies for entering medical students and the SCCT were used to guide the development of the virtual curriculum.^25,30^ Health disparities served as a curricular theme. See Table 2 for a summary of program activities, learning objectives, and example topics.

**Table 2.** The Bronx Health Opportunities Partnership (at) Einstein Virtual Summer Program's Curriculum Design

Program activity	Learning objectives	Example topics/activities
Lectures^a^ Medical specialties and research Disparities in health and health care	• Provide a realistic view of the health care system and health inequities • Understand the value and importance of diverse backgrounds and perspectives in eliminating health disparities • Strengthen knowledge on various health professions and scientific topics in the medical field	A Researcher's Experience Studying COVID-19: protocol, challenges, and possible preliminary findings, Social Determinants of Health, Neurosurgery Career
Workshops	• Improve professional and intrapersonal skills to become competent and self-aware leaders in the health care field • Acquire clinical and study skills practiced in biomedical graduate programs	Mastering the Interview, Reflective Writing/Personal Statement Workshop, Study Strategies, Heart and Lung Sounds
Panel discussions	• Encourage and prepare students from URiM/disadvantaged backgrounds to pursue and apply for a biomedical degree	Einstein Admissions and Financial Aid, Medical Student and Physician Panel
Clinical simulation MECIS sessions^b^	• Provide exposure to clinical scenarios, including patient-physician communication, medical terminology and medical devices • Consider how the social determinants of health affect patient wellness	
Small group sessions	• Obtain mentorship and resources beneficial for biomedical career journeys • Develop networks of peers and near-peer mentors in the biomedical field • Build a professional network	Einstein Medical Student Mentors, Einstein/Montefiore “Talk with a Doc” Sessions
Family evening event^c^	• Provide students and their families with information on how to prepare, apply for and finance a graduate biomedical degree • Address familial concerns about students pursuing a career in the biomedical field	
Student projects	• Apply learned knowledge about health disparities to a medical illness • Exercise time management and oral communication skills	

^a^ Program lectures were conducted on Zoom six to eight times a week and were 45 min in duration.

^b^ We worked with MECIS to create, record, and facilitate two 35–45-min interactive clinical scenarios, including cardiac arrest and childbirth complications. These sessions occurred outside of the regular schedule for groups of six to eight students.

^c^ The event included an MD/PhD panel discussion offered in English and Spanish.

MECIS, Montefiore Einstein Center for Innovation in Simulation; URiM, underrepresented in medicine.

### Mentorship

Thirteen Albert Einstein College of Medicine medical students (eight MD candidates and five MD/PhD candidates) were recruited as near-peer mentors; 12 of the 13 mentors were URiM. Mentors received weekly training sessions before and throughout the program along with a debrief at its conclusion. Each mentor was assigned to a group of six to eight students. There was a total of 11 mentorship sessions and two forums that occurred for 45–60 min. In addition, five out of the six mentor groups met outside of the scheduled sessions at least once a week.

### Evaluation

Program and curricular feedback were obtained through three self-assessment surveys designed to collect quantitative and qualitative responses before and immediately following the virtual program. This study was reviewed by and received Exempt status from the Institutional Review Board of the Albert Einstein College of Medicine. The first pre-program survey, administered in April 2020, collected participants' perspectives on the pandemic, for example, “Considering the current Pandemic, can you share how COVID-19 has personally impacted you?” The second pre-program survey collected students' demographics and career goals, and asked students to rate their confidence and self-efficacy in their ability to achieve goals on a Likert scale. The post-program questionnaire contained identical self-assessment questions to the second survey and included open-ended questions, asking students to describe their experiences in the program and if their expectations were met or exceeded.

### Analysis

#### Quantitative methods

Statistical analysis, which included descriptive statistics, including means and frequencies derived on SPSS v.24, was used to describe the participants. The Fisher Freeman-Halton exact test was conducted to determine the existence of nonrandom associations between the pre- and post-program responses related to self-efficacy, confidence, and so on.^31^

#### Qualitative methods

Free-text responses to the open-ended survey questions were thematically analyzed using inductive thematic analysis.^32^ S.F. and C.S.-H. independently read the text to become familiar with the data before developing codes. S.F. and C.S.-H. met to review and discuss codes and develop preliminary themes inductively, using an iterative process to refine the themes. Nerys Benfield independently reviewed the text and thematic analysis. Discrepancies were reconciled among all team members.

## Results

More than 80% of our participants were URiM; 79% lived in New York and 35% resided in the Bronx. Eighty-three percent were female, and more than 80% were a Junior, Senior or recent graduate, as of Fall 2020. Of the 46 enrolled students, 44 (96%) completed the 6-week program (Table 1).

### Quantitative assessment

Table 3 demonstrates the changes in students' interests in health career disciplines based on pre- and post-program surveys where each respondent was allowed to select multiple health professions of interest. Of the 41 students who completed a pre-program survey, 83% chose physician and 39% chose scientist/researcher, while interest in these careers increased to 93% and 46%, respectively, based on 30 students who completed the post-program survey.

**Table 3.** Career Interests of the Bronx Health Opportunities Partnership (at) Einstein Virtual Summer Program Participants

Career	Pre-survey,^a^ no. (%)	Post-survey,^a^ no. (%)
Physician	34 (82.9)	28 (93.3)
Scientist/researcher	16 (39)	14 (46.7)
Physician assistant	11 (26.8)	3 (10)
Health educator	8 (19.5)	11 (36.7)
Public policy advocate/researcher	7 (17.1)	5 (16.7)
Emergency medical technician	5 (12.2)	7 (23.3)
Psychologist	5 (12.2)	4 (13.3)
Research coordinator	4 (9.8)	5 (16.7)
Nurse practitioner	3 (7.3)	1 (3.3)
Optometrist	3 (7.3)	2 (6.7)
Physical/occupational/speech Therapist	3 (7.3)	2 (6.7)
Registered nurse	2 (4.9)	2 (6.7)
Chiropractor	1 (2.4)	1 (3.3)
Dentist	1 (2.4)	1 (3.3)
Pharmacist	1 (2.4)	1 (3.3)
Social worker	1 (2.4)	0 (0.0)

^a^ Pre–post surveys allowed students to designate >1 profession. Therefore, we did not conduct a statistical test of change. There were 41 pre-survey responses and 30 post-survey responses.

Table 4 provides the distribution of students' responses in the pre- and post-program surveys related to aspects of their self-efficacy measured using a Likert scale. For the question, “I feel informed about what I need to do to reach my goal of becoming a health/science professional,” 39% agreed and strongly agreed on the pre-program questionnaire and 96.6% agreed and strongly agreed on the post-program questionnaire. As for how the students felt about their ability to successfully perform all requirements to achieve their career goals, 58.5% felt very good and excellent on the pre-questionnaire, whereas 93.4% felt very good and excellent on the post-questionnaire. Finally, in the context of students feeling confident in achieving their career goals, 70.8% felt confident and completely confident on the pre-questionnaire and 87.2% felt confident and completely confident on the post-questionnaire. The *p*-value from the Fisher Freeman-Halton exact test was statistically significant (<0.05) for five of the six survey questions, suggesting nonrandom associations in the change between the students' pre- and post-program responses (Table 4).

**Table 4.** Statistical Analysis of Students’ Self-Efficacy Changes Before and After the Bronx Health Opportunities Partnership (at) Einstein Virtual Summer Program

	Student likert responses on pre–post surveys, no. (%)						Pre–post change: Fisher-Freeman-Halton, Exact test	
Question	Strongly disagree	Disagree	Unsure	Agree	Strongly agree	Total	Value	p^a^
1. I feel informed about what I need to do to reach my goal of becoming a health/science professional.								
Pre	2 (4.9)	6 (14.6)	17 (41.5)	15 (36.6)	1 (2.4)	41	36.708	**0.000**
Post	0 (0.0)	0 (0.0)	1 (3.3)	13 (43.3)	16 (53.3)	30		
2. I feel confident in my ability to network.								
Pre	3 (7.3)	9 (22.0)	15 (36.6)	10 (24.4)	4 (9.8)	41	20.905	**0.000**
Post	0.(0.0)	0 (0.0)	4 (13.3)	17 (56.7)	9 (30.0)	30		
3. I feel prepared on how to present myself during an interview.								
Pre	1 (2.4)	10 (24.4)	10 (24.4)	13 (31.7)	7 (17.1)	41	15.333	**0.002**
Post	0 (0.0)	0 (0.0)	3 (10.0)	16 (53.3)	11 (36.7)	30		
4. I feel prepared on how to write a resume.								
Pre	0 (0.0)	5 (12.2)	12 (29.3)	12 (29.3)	12 (29.3)	41	11.137	**0.008**
Post	0 (0.0)	0(0.0)	2 (6.7)	16 (53.3)	12 (40.0)	30		
5. I feel confident in my verbal and written communications.								
Pre	2 (4.9)	4 (9.8)	13 (31.7)	15 (36.6)	7 (17.1)	41	6.648	0.127
Post	0 (0.0)	1 (3.3)	4 (13.3	17 (56.7)	8 (26.7)	30		

	Not confident	Slightly confident	Somewhat confident	Confident	Completely confident	Total	Value	p
6. How confident do you feel in your ability to successfully perform all of the tasks and activities required to achieve your health science career goal?								
Pre	1 (2.4)	1 (2.4)	15 (36.6)	14 (34.1)	10 (24.4)	41	12.016	**0.007**
Post	0 (0.0)	0 (0.0)	2 (6.7)	20 (66.7)	8 (26.7)	30		
7. How confident are you that you will achieve your career goals?								
Pre	1 (2.4)	2 (4.9)	9 (22.0)	17 (41.5)	12 (29.3)	41	3.110	0.582
Post	0 (0.0)	1 (3.3)	3 (10.0)	17 (56.7)	9 (30.5)	30		

^a^ Statistically significant *p*-values (*p*<0.05) are displayed in bold.

### Qualitative assessment

Five major qualitative themes emerged from our analysis of students' responses to open-ended questions on the pre- and post-program evaluation surveys. The first two themes and subthemes described the students' perspectives on the impact of the pandemic in April 2020, 1 month after the national implementation of social distancing and quarantine mandates. Students' described how moving off campus and remote learning left gaps in’ social and academic supports, yielding Theme (1), “loss of a supportive environment,” and its consequent subtheme (1.1), “the challenging learning environment.” Theme (2), “derailed or uncertain future,” expressed how the cancellations and postponements of planned or promised career opportunities left students unsure about their career timelines. Subtheme (2.1), “COVID-19 hitting home,” detailed the personal impact of the illness, including students' fears and concerns about their families' and their own futures.

Analysis of students' responses following the virtual program resulted in the following themes: (3) “Supportive Community,” in which students described the meaningful and supportive connections made with mentors, peers, and faculty in the virtual program and how this affected their career-self-efficacy and outcome expectations; and (4) “Inspired and Reinforced Goals,” where students shared how personal program experiences motivated their career interests and choice goals. Themes were connected back to the SCCT. The virtual program appears to have provided learning experiences along with proximal support, which impacted students' career-related goals, outcome expectations, and choices.^27^

#### Theme 1: Loss of Supportive Environment

Students described how the pandemic interrupted access to the support they previously received on campus, including tutoring, study spaces, and services for students with learning and other disabilities. “As someone that has a learning disability I rely heavily on the in person interactions with the professors.” Many URiM and disadvantaged college students rely on their school's facilities and resources well beyond academics.^33^ Campus closures were announced in the middle of the Spring 2020 semester, leaving students with little time to adjust, “Having to abruptly move off of campus was difficult for me, as I depend on school for housing and usually stay on campus even during breaks.” They used the words “panic” and “chaos” to describe the sudden move from campus. Social interaction and support from friends and classmates were also missed by students during the quarantine. “Before the current pandemic, I felt as though I had a very strong support system…” Sheltering-in-place, students reported feeling “isolated” and “distanced from family and friends.”

#### Subtheme 1.1: Challenging Learning Environment

My performance in a …. required prehealth course went downhill and I had to drop the class on recommendation from the professor. I will not be able to take the class again.

Remote learning was stressful for many students, especially for those enrolled in “gateway” and advanced science courses. “I was still taking science classes which was harder to learn online since the material is better taught in person.” Many students described remote learning as “hard; difficult” and most reported that online learning had a negative impact on their performance and grades, including the pass/fail option implemented by most colleges. “All classes [were] mandatory pass fail and not being counted [towards my] GPA.” In addition to issues with adapting to remote learning, students had trouble finding suitable learning/study spaces at home. “I don't have a quiet place to study due to moving off campus.” Others had to take on more responsibilities at home to help their working family members. “Moving back home…required me to take on home responsibilities like babysitting. I have had to multi-task when doing coursework… and push back studying for classes/MCAT.”

#### Theme 2: Derailed or Uncertain Future

Our students described derailed plans and uncertain futures: “COVID-19 has … disrupted my plans to volunteer at a medical facility, employment, and to travel for educational opportunities.” This theme of interruption and uncertainty echoed throughout the student responses with stories of lost opportunities, “…not being able to shadow this summer as planned, not working in the lab I was set to go to, and the loss of a promised publication.” They expressed feeling derailed from their career paths and unsure about their futures. Students described how their future's ambiguity also impacted their MCAT plans, including changing or delaying their examination date, “Hav[e] to move up [my] MCAT to avoid uncertainties and stress if my university changes something mid year, so less study time.”

#### Subtheme 2.1: COVID-19 Hitting Home

Students had personal experiences with COVID-19 that added more uncertainty about their families and their own well-being. These experiences included contracting COVID-19 themselves, as well as the illness and/or death of others. “Above all else it really hurt to lose individuals I cared about.” COVID-19 deaths revealed to be the most prominent in disadvantaged communities, which most of our students belong to.^22^ The real and perceived pressure to stay on track academically offered no time or space to cope with loss, which was often sudden and traumatic.

My family had a passing of a loved one which took [us] by surprise. I had to work very hard to stay mentally strong while I completed my courses as this all happened at the same time.

Many lived in close quarters with sick family members and worried about contracting the virus, “currently at risk for COVID-19 because a family member in my house is likely positive.” Minority and low-income groups are disproportionately represented in frontline work, considered essential workers, and, therefore, at increased risk of contracting COVID-19.^34^ “My father has continued to work throughout quarantine, but he has recently fallen ill…. he is the sole source of income for this household.” Financial concerns and fears were common, as most of our students are low-income and do not have the financial cushion to withstand a loss of income. Working students worried about how they would pay tuition after their workplaces closed and they were furloughed. “I wanted to work this summer to help pay for my tuition next semester, but no one is hiring during the pandemic.”

#### Theme 3: Supportive Community

Through the virtual program, students felt connected to peers and mentors who provided socioemotional and instrumental support that they lacked. “I have not had a good relationship with a mentor until this program.”

Although virtual, I was able to be [a part] of a community of incredible people who I will have for the rest of my school career and beyond.

Students described their impressions of their mentors as “caring” and “genuine.” Mentors provided students with reassurance and advice on dealing with insecurities such as “imposter syndrome” and fears of failure, along with other socioemotional support offered by mentors sharing their own personal stories: “I was able to learn what medical school is like from my mentors sharing their experiences—particularly…..overcoming racism and sexism.” Many mentors shared books, study materials, and other resources for courses and offered to review secondary medical school applications. Students made meaningful relations with mentors as well as peers who they were “able to get close with … and still form a connection over Zoom,” and felt a sense of belonging, “I am not alone while on my pre health track,” which they greatly valued.

#### Theme 4: Inspired or Reinforced Goals

Students valued opportunities to meet and speak with physicians and learn about health disparities through lectures and clinical simulations. “I [met] a physician who inspired me even more to pursue medicine.” For many, this reinforced their career goals, “… solidified my reasoning in wanting to become a physician” or assisted in their decision-making process, providing a “clearer idea of the career I want to pursue (MD/MPH).” Many of the health professionals were URiM and/or women; students found this inspiring, “I was able to meet doctors who [were] the same color [as me]!” For URiM students who rarely see clinicians and researchers of color, meeting URiM professionals can send the message that they, too, can achieve a career in science and health care and that they do belong in the field. Students valued the health disparities topics and felt “motivated” to pursue careers in biomedicine. They were also inspired by how they could be instrumental in achieving health equity through practice and advocacy by providing “a voice for those [we] serve.”

## Discussion

Results of our pre- and post-program surveys demonstrate a significant increase in students' career self-efficacy following the program, in most areas addressed by the curriculum, including feeling informed about what they needed to do to reach their career goals, confidence in their ability to network, and prepared on how to present themselves during an interview and how to write a resume, along with confidence in their ability to perform all the tasks required to achieve their career goals. These findings are consistent with the SCCT. However, there was no significant pre- to post-program increase in confidence for verbal and written communication, which might be expected since we did not include written communication as a curricular objective. Furthermore, no significant pre- to post-program increase was observed for confidence in achieving career goals, although a different version of this question did yield significant increase from pre- to post-program. These discrepancies are further explained in the Limitations section. Our results suggest that we were effectively able to meet the curricular objectives designed for our in-person program within a virtual setting.

Our findings also revealed that the existing disadvantages URiM students experience were compounded by COVID-19. Aucejo et al. found that “…the economic and health related shocks induced by COVID-19 vary systematically by socioeconomic factors and constitute key mediators in explaining the large (and heterogeneous) effects of the pandemic [on undergraduate students' educational outcomes].”^35^ These factors added to the environmental barriers faced by URiM and disadvantaged students on their paths to biomedical careers. The results of this study, exploring the impact of COVID-19 on the student's career trajectory, are in direct alignment with the SCCT and clearly demonstrate the triadic relationship between self-concept, environment, and external factors. When asked about how COVID-19 has impacted them, two themes emerged: loss of supportive environment and derailed or uncertain future, with the subthemes of challenging learning environment and COVID-19 hitting home. The loss of supportive environment speaks directly to the students' change from onsite and direct access to supports to remote learning and interaction (environment) due to COVID-19 (external factors). This change in the learning environment impacted the student's feelings and thoughts (self-concept) about their future, resulting in uncertainty and concern (Theme 2: derailed or uncertain future). College campuses provide a much-needed home base for disadvantaged students, including community and resources.^33^ Therefore, the closure of universities and abrupt movement to online learning deepened students' challenges toward access to supportive services and resources, such as campus jobs. Furthermore, the cancellation of clinical shadowing, research, and summer enrichment opportunities brought upon a summer of isolation and distance from their premedical goals.

First-generation college students and those without access to biomedical professionals within families and social networks often become discouraged, while attempting to pursue their dreams in the absence of additional information and support.^9,13,36^ Even well-meaning family members can be a source of unintended discouragement when they are not aware of or able to support students on such an unfamiliar path. The loss of family members may have also magnified preceding family stress and inability to provide a supportive environment.

Students' experiences with COVID-19 illness and death illustrated in the COVID-19 hitting home theme reflected the increased health and financial vulnerability of our students and their families, consistent with the literature on COVID-19 in minority populations. Chetty et al. reported that employment rates fell by 37% at the start of the COVID-19 recession (April 2020) for low-wage workers compared to 14% for those in the top-wage quartile; employment levels for workers in the top-wage quartile returned to pre-COVID levels by the end of May, while remaining 20% below baseline for low-wage workers as of October 2020.^23^

The Bronx HOPE Virtual Summer Program was developed to address the need for continued support and vicarious learning, and to positively influence outcome expectations among URiM students in a time of disruption and uncertainty. This program offered an interactive, 6-week, virtual summer experience that incorporated lectures, workshops, and multilevel mentoring. Students' responses on the post-intervention survey also demonstrate the triadic relationship and immense impact that virtual interventions with supportive mentorship can have on self-concept and career choice development. The themes that emerged were supportive community and inspired or reinforced goals, with students stating this experience helped them to develop clearer goals and a deeper understanding as to why a career in medicine was right for them. In addition, through the near-peer mentorship components, students were paired with medical students who are from similar backgrounds and cultures, which impacted their level of self-efficacy and belief that if “someone like them” could make it, maybe they could too.

### Limitations

This evaluation included a small sample of undergraduate students, mostly living in New York, which may make the results less generalizable to all URiM undergraduate students. We used anonymous surveys. Therefore, we could not determine who was responding or match pre–post data for the analysis of individual change. This was intentional, as we wanted to collect students' honest responses and minimize social desirability pressures. Fourteen students did not complete the post-program survey and, therefore, we are not able to determine if response bias may have caused participants with more positive experiences to be more likely to respond. However, our retention rate was over 96% and the average session attendance was 89%, suggesting that most students found value in the program. On the last day of the program, many students were moving back into their dorms and, therefore, may not have completed the post-program survey. Our survey included a learning outcome that was not included in the virtual curriculum, written communication, and there was redundancy in two questions assessing confidence in achieving career goals. While the use of surveys to collect responses may have limited the depth of the perspectives gathered, our findings indicate that students' biomedical career goals and confidence were reinforced or strengthened following participation in the virtual program. A longitudinal study would allow for an evaluation of more distal career-related outcomes.

## Conclusions

The COVID-19 pandemic is a major educational disruptor, especially for URiM and other underserved students who face challenges and obstacles that are often invisible to most. In general, virtual programs are challenging to implement and are usually perceived as disconnected and isolating, but under current circumstances, these challenges are magnified, bringing additional feelings of chaos, panic, and uncertainty. To reduce the risk of COVID-19 transmission, remote learning was a clear necessity, but its implementation could be improved with the inclusion of more socioemotional supports. Our findings suggest that connectedness and community can be created within virtual environments, which leads to increased self-efficacy, a 30% increase within our study population, and motivation to persevere.

It is important to note that our program activities occurred during a time of a major social justice movement, following the death of George Floyd at the hands of police. Although we did not collect students' perspectives related to the impact of these events on our surveys, we acknowledge that this might have compounded the stress and uncertainty students were already experiencing. We note that major “data-driven” philanthropies to “improve the health and wealth” of communities of color have targeted increasing the number of URiM physicians to “save lives… and reduce health problems that limit economic opportunities” in these communities.^37^ We advocate for the social justice represented by more of such “pipeline” programs, exemplified by expanding the federal Centers of Excellence and Health Careers Opportunity Programs,^1,2,7^ which launched Bronx HOPE, as well as comprehensive and long-term evaluations to measure the impact of educational policies and practices within the most vulnerable groups to determine the appropriate supports required to achieve equitable access to education, health, and wealth.

## Abbreviations Used

HOPE: Health Opportunities Partnership (at) Einstein
MECIS: Montefiore Einstein Center for Innovation in Simulation
SCCT: Social Cognitive Career Theory
URiM: underrepresented in medicine

## References

[1] Smedley BD, Butler AS, Bristow LR; Institute of Medicine (U.S.), Committee on Institutional and Policy-Level Strategies for Increasing the Diversity of the U.S. Health Care Workforce, Institute of Medicine (U.S.), Board on Health Sciences Policy*. In the Nation's Compelling Interest: Ensuring Diversity in the Health-Care Workforce*. Washington, DC: National Academies Press, 2004 25009857

[2] Evans M. Healthcare's minority report. Sullivan Commission, IOM try to make patient, hospital staff makeup more reflective of the nation's ever-changing population. Mod Healthc. 2004;34:6–7, 14, 1. 15506502

[3] Alsan M, Garrick O, Graziani G. Does diversity matter for health? Experimental evidence from Oakland. Am Econ Rev. 2019;12:4071–4111.

[4] Lett LA, Murdock HM, Orji WU, et al. Trends in racial/ethnic representation among US Medical Students. JAMA Netw Open. 2019;2:e1910490. 31483469 10.1001/jamanetworkopen.2019.10490 PMC6727686

[5] Diversity in medicine: Facts and figures 2019. 2019. Available at https://www.aamc.org/data-reports/workforce/interactive-data/figure-6-percentage-acceptees-us-medical-schools-race/ethnicity-alone-academic-year-2018-2019 Accessed September 5, 2020.

[6] Alexander C, Chen E, Grumbach K. How leaky is the health career pipeline? Minority student achievement in college gateway courses. Acad Med. 2009;84:797–802. 19474563 10.1097/ACM.0b013e3181a3d948

[7] Grumbach KCJ, Gandara P, Munoz C, et al. Strategies for Improving the Diversity of the Health Professions: Center for California Health Workforce Studies. San Francisco, CA: UCSF; Education Policy Center, UC Davis; California Endowment, 2003.

[8] Barr DA, Gonzalez ME, Wanat SF. The leaky pipeline: factors associated with early decline in interest in premedical studies among underrepresented minority undergraduate students. Acad Med. 2008;83:503–511. 18448909 10.1097/ACM.0b013e31816bda16

[9] Camacho A, Zangaro G, White KM. Diversifying the health-care workforce begins at the pipeline: a 5-year synthesis of processes and outputs of the scholarships for Disadvantaged Students Program. Eval Health Prof. 2017;40:127–150. 26660346 10.1177/0163278715617809

[10] Fritz CD, Press VG, Nabers D, et al. SEALS: an Innovative Pipeline Program targeting obstacles to diversity in the Physician Workforce. J Racial Ethn Health Disparities. 2016;3:225–232. 27271062 10.1007/s40615-015-0131-x

[11] Derck J, Zahn K, Finks JF, et al. Doctors of tomorrow: an innovative curriculum connecting underrepresented minority high school students to medical school. Educ Health (Abingdon). 2016;29:259–265. 28406112 10.4103/1357-6283.204219

[12] Crews DC, Wilson KL, Sohn J, et al. Helping scholars overcome socioeconomic barriers to medical and biomedical careers: creating a pipeline initiative. Teach Learn Med. 2020;32:422–433. 32096414 10.1080/10401334.2020.1729161

[13] Smith SG, Nsiah-Kumi PA, Jones PR, Pamies RJ. Pipeline programs in the health professions, part 1: preserving diversity and reducing health disparities. J Natl Med Assoc. 2009;101:836–840, 45–51. 19806840 10.1016/s0027-9684(15)31030-0

[14] Winkleby MA. The Stanford Medical Youth Science Program: 18 years of a biomedical program for low-income high school students. Acad Med. 2007;82:139–145. 17264691 10.1097/ACM.0b013e31802d8de6

[15] Kana LA, Noronha C, Diamond S, et al. Experiential-learning opportunities enhance engagement in Pipeline Program: a qualitative study of the Doctors of Tomorrow Summer Internship Program. J Natl Med Assoc. 2020;112:15–23. 32037249 10.1016/j.jnma.2019.11.006

[16] Howell LP, Wahl S, Ryan J, et al. Educational and career development outcomes among Undergraduate Summer Research Interns: a pipeline for pathology, laboratory medicine, and biomedical science. Acad Pathol. 2019;6:2374289519893105. 31858021 10.1177/2374289519893105 PMC6913060

[17] Stephenson-Hunter C, Adames TR, Franco S, et al. Analysis of the curriculum of a Summer Pipeline Program for economically disadvantaged premedical students in the Bronx, NY. J Best Pract Health Prof Divers. 2019;12:1–23.

[18] 2020 County health rankings report: New York. 2020. Available at https://www.countyhealthrankings.org/reports/state-reports/2020-new-york-report Accessed April 13, 2021.

[19] COVID-19: data. 2020. Available at https://www1.nyc.gov/site/doh/covid/covid-19-data.page Accessed April 13, 2021.

[20] NYCDOHMH, Data by Borough. 2021. Available at https://www1.nyc.gov/site/doh/covid/covid-19-data-totals.page#boro Accessed April 15, 2021.

[21] Magnani JW, Kinloch V, Essien UR. Separate and unequal: the cost of coronavirus disease 2019 on childhood health and well-Being. Health Equity. 2021;5:72–75. 33681692 10.1089/heq.2020.0080 PMC7929920

[22] Alcendor DJ. Racial disparities-associated COVID-19 mortality among minority populations in the US. J Clin Med. 2020;9:2442. 10.3390/jcm9082442 PMC7466083 32751633

[23] Chetty R, Friedman JN, Hendren N, Stepner M. The economic impacts of COVID-19: evidence from a new public database built using private sector data. National Bureau of Economic Research Working Paper Series. 2020:27431. 10.1093/qje/qjad048 PMC11189622 38911676

[24] Bandura A. Self-efficacy: toward a unifying theory of behavioral change. Psychol Rev. 1977;84:191. 847061 10.1037//0033-295x.84.2.191

[25] Lent RW, Brown SD, Hackett G. Toward a unifying social cognitive theory of career and academic interest, choice, and performance. J Vocat Behav. 1994;45:79–122.

[26] Lent RW, Brown SD. Social cognitive model of career self-management: toward a unifying view of adaptive career behavior across the life span. J Couns Psychol. 2013;60:557–568. 23815631 10.1037/a0033446

[27] Lent RW, Morris TR, Penn LT, Ireland GW. Social-cognitive predictors of career exploration and decision-making: longitudinal test of the career self-management model. J Couns Psychol. 2019;66:184–194. 30091621 10.1037/cou0000307

[28] Lent RW, Lopez FG, Brown SD, Gore PA Jr. Latent structure of the sources of mathematics self-efficacy. J Vocat Behav. 1996;49:292–308. 8980086 10.1006/jvbe.1996.0045

[29] Lent RW, Sheu HB, Miller MJ, et al. Predictors of science, technology, engineering, and mathematics choice options: a meta-analytic path analysis of the social-cognitive choice model by gender and race/ethnicity. J Couns Psychol. 2018;65:17–35. 29355343 10.1037/cou0000243

[30] Core Competencies for Entering Medical Students. ND. Available at https://students-residents.aamc.org/applying-medical-school/article/core-competencies Accessed January 20, 2021.

[31] Freeman GH, Halton JH. Note on an exact treatment of contingency, goodness of fit and other problems of significance. Biometrika. 1951;38:141–149. 14848119

[32] Braun V, Clarke V. Using thematic analysis in psychology. Qual Res Psychol. 2006;3:77–101.

[33] Sahu P. Closure of Universities Due to Coronavirus Disease 2019 (COVID-19): impact on education and mental health of students and academic staff. Cureus. 2020;12:e7541. 32377489 10.7759/cureus.7541 PMC7198094

[34] Kalyanaraman Marcello R, Dolle J, Grami S, et al. Characteristics and outcomes of COVID-19 patients in New York City's public hospital system. PLoS One. 2020;15:e0243027. 33332356 10.1371/journal.pone.0243027 PMC7745980

[35] Aucejo EM, French J, Ugalde Araya MP, Zafar B. The impact of COVID-19 on student experiences and expectations: evidence from a survey. J Public Econ. 2020;191:104271. 32873994 10.1016/j.jpubeco.2020.104271 PMC7451187

[36] Upshur CC, Wrighting DM, Bacigalupe G, et al. The Health Equity Scholars Program: innovation in the Leaky Pipeline. J Racial Ethn Health Disparities. 2018;5:342–350. 28526974 10.1007/s40615-017-0376-7 PMC5694706

[37] De la Merced MJ, Sorkin AR. Michael Bloomberg Gives $100 Million to Historically Black Medical Schools. The New York Times, 2020.

